# Letter from the Program Directors

**DOI:** 10.19102/icrm.2021.120401

**Published:** 2021-04-15

**Authors:** Wendy Tzou, William Sauer


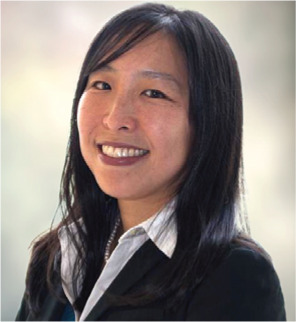
 
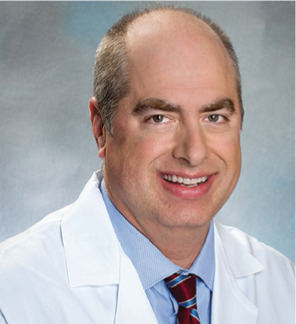


Dear readers,

As the Program Directors for the Electrophysiology Fellows Summit, we are proud to introduce the published case reports of the three finalists who were selected to present their unique cases during the Fellows and Residents Case Competition session during the virtual Electrophysiology Fellows Summit in December 2020.

After a meticulous review of the numerous exceptional case entries submitted by fellows and residents from around the globe, the program committee nominated these finalists to present their work and participate in panel discussions, with the overall winner announced at the sessions’ conclusion following the panel deliberation. (For those who were unable to attend or who wish to view the sessions again, EP Fellows Summit ON-DEMAND at www.epfellowssummit.com provides instant and unlimited access to the full library of educational programming from the Summit).

First, Dr. Andrew Locke of Beth Israel Deaconess Medical Center in Boston, MA presents a case of superior vena cava syndrome in a young woman with arrhythmogenic cardiomyopathy due to a plakophilin-2 mutation and previous single-chamber implantable cardioverter-defibrillator placement for secondary prevention purposes. The title of this case understates the impressive spectrum of complex issues that were managed in a single patient, who had more than just lead-related superior vena cava syndrome. Presented in an eloquent fashion and outlining a systematic approach, this case report exemplifies the complexity of many of our patients, in whom there is not an isolated teaching point but rather a variety of important learning opportunities. As the case competition winner, Dr. Locke’s case report is complemented by a brief expert commentary from his mentor, Dr. Peter Zimetbaum, Associate Chief and Clinical Director of Cardiology at Beth Israel Deaconess Medical Center, highlighting the significance of the case.

Next, Dr. Yasuhito Kotake in the Cardiology Department at Westmead Hospital in Westmead, Australia presents a case of epicardial–endocardial reentry in a 53-year-old man with ischemic cardiomyopathy, ventricular tachycardia, and previous implantable cardioverter-defibrillator placement. Increasingly recognized and beautifully exemplified in this case report is the three-dimensionality in ventricular tachycardia substrates and circuits in structural heart disease. While this is a well-recognized phenomenon in nonischemic ventricular tachycardia, this phenomenon should also be respected and considered in postinfarction ventricular tachycardias, especially in the area of early revascularization, in which mixed substrates appear to be more prevalent.

Finally, Dr. Leah John of the Medical University of South Carolina in Charleston, SC presents the case of a young woman with cardioinhibitory vasovagal syncope who underwent cardioneural ablation. An emerging and potentially revolutionary technique for alleviating cardioinhibitory vasovagal syncope, potentially deferring the need for pacemaker implantation, is refreshingly presented in this case report. Perhaps more importantly, however, John and colleagues highlight how essential creative problem-solving and collaboration are in sustaining lifelong learning and continuing advances in our field.

Congratulations to Dr. Locke on his winning case and to Drs. John and Kotake as the case competition co-finalists for their unique case presentations.

We look forward to your attendance at this year’s Electrophysiology Fellows Summit, scheduled from December 10 to 11, 2021. As a hybrid conference, attendees will have the choice of attending the Summit virtually or as a traditional in-person event in San Antonio, TX, with the chance to participate in hands-on training sessions and interpersonal engagement, enjoy the sights and sounds of the San Antonio Riverwalk, and do much more. For those who are unable to attend the Summit in San Antonio, virtual attendance and engagement will be made possible from the convenience of your computer or mobile device through the Summit’s innovative livestreaming broadcast platforms. Detailed information will be available at www.epfellowssummit.com.

Sincerely,

Wendy Tzou, md

University of Colorado Anschutz Medical Campus

Aurora, CO, USA

and

William Sauer, md

Brigham and Women’s Hospital

Boston, MA, USA

